# E4orf1: A Novel Ligand That Improves Glucose Disposal in Cell Culture

**DOI:** 10.1371/journal.pone.0023394

**Published:** 2011-08-23

**Authors:** Emily J. Dhurandhar, Olga Dubuisson, Nazar Mashtalir, Rashmi Krishnapuram, Vijay Hegde, Nikhil V. Dhurandhar

**Affiliations:** Infections and Obesity Laboratory, Pennington Biomedical Research Center, Louisiana State University System, Baton Rouge, Louisiana, United States of America; University of Las Palmas de Gran Canaria, Spain

## Abstract

Reducing dietary fat intake and excess adiposity, the cornerstones of behavioral treatment of insulin resistance(IR), are marginally successful over the long term. Ad36, a human adenovirus, offers a template to improve IR, independent of dietary fat intake or adiposity. Ad36 increases cellular glucose uptake via a Ras-mediated activation of phosphatidyl inositol 3-kinase(PI3K), and improves hyperglycemia in mice, despite a high-fat diet and without reducing adiposity. Ex-vivo studies suggest that Ad36 improves hyperglycemia in mice by increasing glucose uptake by adipose tissue and skeletal muscle, and by reducing hepatic glucose output. It is impractical to use Ad36 for therapeutic action. Instead, we investigated if the E4orf1 protein of Ad36, mediates its anti-hyperglycemic action. Such a candidate protein may offer an attractive template for therapeutic development. Experiment-1 determined that Ad36 ‘requires’ E4orf1 protein to up-regulate cellular glucose uptake. Ad36 significantly increased glucose uptake in 3T3-L1 preadipocytes, which was abrogated by knocking down E4orf1 with siRNA. Experiment-2 identified E4orf1 as ‘sufficient’ to up-regulate glucose uptake. 3T3-L1 cells that inducibly express E4orf1, increased glucose uptake in an induction-dependent manner, compared to null vector control cells. E4orf1 up-regulated PI3K pathway and increased abundance of Ras–the obligatory molecule in Ad36-induced glucose uptake. Experiment-3: Signaling studies of cells transiently transfected with E4orf1 or a null vector, revealed that E4orf1 may activate Ras/PI3K pathway by binding to *Drosophila* discs-large(Dlg1) protein. E4orf1 activated total Ras and, particularly the H-Ras isoform. By mutating the PDZ domain binding motif(PBM) of E4orf1, Experiment-4 showed that E4orf1 requires its PBM to increase Ras activation or glucose uptake. Experiment-5: In-vitro, a transient transfection by E4orf1 significantly increased glucose uptake in preadipocytes, adipocytes, or myoblasts, and reduced glucose output by hepatocytes. Thus, the highly attractive anti-hyperglycemic effect of Ad36 is mirrored by E4orf1 protein, which may offer a novel ligand to develop anti-hyperglycemic drugs.

## Introduction

The United States alone has about 26 million individuals with diabetes mellitus (DM), and even more (79 million) with prediabetes (PreDM) (National Diabetes Factsheet released by the Centers for Disease Control http://www.cdc.gov/diabetes/pubs/pdf/ndfs_2011.pdf). Better drugs are urgently needed to treat hyperglycemia and associated comorbidities. Most anti-diabetic agents yield better results, if combined with behavior modification to reduce dietary fat intake and obesity. However, despite their obvious health benefits, long term compliance with such behavioral changes is highly challenging for the general population. Therefore, agents to improve insulin resistance independent of adiposity or dietary fat intake would be extremely attractive and of *practical benefit*.

Our recent data indicate that Ad36, a human adenovirus, may offer a template to develop such an agent. In humans, natural Ad36 infection predicts better glycemic control independent of age, sex or adiposity [1]. Experimental Ad36 infection of mice improves hyperglycemia, despite a 60% fat diet and without reducing adiposity [1]. Signaling studies suggest that in these mice, Ad36 improves glycemic control by increasing glucose uptake by adipose tissue and skeletal muscle and by reducing hepatic glucose output [1]. Mechanistic *in vitro* studies show that Ad36 increases insulin independent glucose uptake in diabetic and non-diabetic human adipose tissue explants [2] and in human primary muscle cell culture in a dose dependent manner [3]. Ad36 requires Ras mediated activation of phosphatidyl inositol 3-kinase (PI3K), to increase cellular glucose uptake [2], [3].

Collectively, these data reveal anti-hyperglycemic properties of Ad36,that are clinically relevant to humans, and offer a tool to develop new anti-diabetic agents. Harnessing certain properties of viruses for beneficial purposes has been creatively used for several years. For example, anti-bacterial properties of bacteriophage virus [4], the oncolytic ability of a mutant adenovirus [5], or the use of Herpes simplex virus and several other viruses for lysing cancer cells [6], have been used alone, or with various synergistic drugs [7], [8]. Clearly, infection with Ad36 is not a viable treatment option. Instead, identifying the viral protein responsible for Ad36-induced glucose disposal is the next step in harnessing the anti-hyperglycemic potential of the virus for therapeutic purpose.

Adenoviruses have a set of several early genes that encode proteins for evading the host immune system and changing cell function for favorable viral replication, and several late genes, that encode structural proteins required for viral particle assembly. This study identified the Ad36 gene that mediates the glucose disposal induced by the virus. Considering that Ad36 requires Ras/PI3K pathway for enhancing glucose uptake [2], [3], we focused on E4orf1 gene of the virus that up-regulates this pathway [9]. The E4orf1 gene of Ad36 is transcribed from the first open reading frame of Ad36 early gene 4, and yields a 17 kDa, 125 amino acid protein, and has a PBM through which it interacts with other proteins containing PDZ regions for scaffolding [9]. E4orf1 is necessary and sufficient for Ad36 to activate the PI3K pathway [9], and its PBM is required for the effect. E4orf1 protein of Ad9, a closely related adenovirus, stimulates Ras-mediated PI3K activation [10], [11], via the interaction of its PBM with Dlg1 protein. In Ad36 infected animals, E4orf1 is expressed in adipose tissue or livers [1], [12], and its expression in the liver positively correlates with glycemic improvement in mice [1]. This background provided the rationale to test the role of E4orf1 as the mediator of Ad36-induced glucose uptake, as outlined below.

By knocking down E4orf1 gene expression in Ad36-infected cells, Experiment 1 determined that Ad36 ‘requires’ its E4orf1 protein for up-regulating cellular glucose uptake. Next, by inducibly expressing only E4orf1 in cells, Experiment 2 identified E4orf1 as ‘sufficient’ to up-regulate the Ras pathway and glucose uptake. Experiment 3 revealed that similar to the action of E4orf1 of Ad9, Ad36 E4orf1 may activate Ras by binding to Dlg1 protein. Moreover, total Ras and particularly, the H-Ras isoform is significantly increased and activated by Ad36 E4orf1. By mutating the PBM of Ad36 E4orf1, Experiment 4 showed that E4orf1 requires its PBM to activate Ras or to increase glucose uptake. Finally, Experiment 5 determined that transient transfection by E4orf1 significantly increases glucose uptake in preadipocytes, adipocytes, and myoblasts, and significantly reduces glucose output by hepatocytes.

## Methods

Outline of each experiment is described below, followed by a detailed description of the techniques and assays (T&A) used. We employed multiple approaches, including siRNA knockdown of Ad36 E4orf1, transient or stable transfection of cells with E4orf1, or constitutive expression of E4orf1 or its mutant, to test the role of E4orf1 in glucose uptake. To pull down proteins, or to test the response of different cell types, V-5 tagged E4orf1 plasmid transfection was used. To obtain large amount of protein required to determine the activation of Ras and its isoforms, a preadipocyte cell line with stable, inducible expression of E4orf1 was used. Finally, cells that constitutively express E4orf1 were used to confirm if glucose uptake is attributed to intact E4orf1 and abrogated by mutating the protein. Specific experiments are described below.

### Experiment 1: Does Ad36 ‘require’ E4orf1 for glucose uptake?

3T3-L1 preadipocytes were plated in 12-well plates and transfected the next day using Lipofectamine (2 µL/well) (Invitrogen, #18324-012) with 100 pmol of either E4orf1 siRNA (Ambion #AM16100) or non-targeting (NT) siRNA (Thermo Scientific, #D-001810-02-05). Immediately upon transfection, the E4orf1 siRNA transfected cells were infected with Ad36 (5 PFU per cell) or mock infected with media. The NT siRNA transfected cells were infected with Ad36. Mock-infected cells with E4orf1 siRNA served as a baseline control, and Ad36 infected cells with either NT siRNA, or with E4orf1 siRNA were the two experimental groups. Two-days post transfection/infection, basal 2-Deoxy-D-glucose (2DG) uptake was determined as described in T&A. This experiment was repeated three times with statistically significant results. Representative data are presented.

### Experiment 2: Is E4orf1 ‘sufficient’ to up-regulate cellular glucose uptake? Does E4orf1 up-regulate the Ras pathway?

A 3T3-L1 cell line that stably expresses E4orf1 in response to doxycycline induction was developed as described in T&A. A TetOn system was used to clone in either pTRE-TIGHT (Null vector control; pTRE cells) or pTRE-E4orf1-TIGHT (3T3-E4 cells). Basal 2DG uptake was determined 24 h post induction in the presence of increasing doses of doxycycline (Clonetech, #631311). Protein was harvested for determining Ras, AKT phosphorylation (as an indicator of PI3K activation), and the abundance of Glut4, Glut1 and adiponectin, by WB.

### Experiment 3: Does E4orf1 interact with Dlg1 and activate Ras?

#### E4orf1 interaction with Dlg1

HEK293 cells were co-transfected with plasmid expressing Dlg1 (GW1-CMV-HA-Dlg1) and either one expressing E4orf1 (pcDNA-V5-Ad36 E4orf1) or a control plasmid (pcDNA-V5-DEST) (Invitrogen #12489-019). Two days post transfection, protein was harvested and immunoprecipitated with HA-antibody (Santa Cruz, #sc-7392). E4orf1 was detected using WB with anti-V5 antibody (Invitogen #46-0705) following the pull down.

#### Activation of total and isoform-specific Ras

To determine isoform-specific activation of Ras, the 3T3-E4 and Null pTRE inducible stable cell lines were induced for 24 hours with 1,000 ng/mL doxycycline, and the activation was detected via a pull down assay for the Ras binding domain (RBD) of Raf, and detected by WB with total and isoform specific antibodies against H-Ras, N-Ras or K-Ras.

### Experiment 4: Does E4orf1 require its PBM for glucose uptake and Ras activation?

#### Ras activation

3T3-L1 cells were transfected with V-5 tagged plasmids expressing intact E4orf1 (pcDNA-V5-Ad36 E4orf1), plasmid expressing E4orf1 with a mutated PBM (Ad36 E4orf-1ΔPBM-V5-pcDNA) or a null vector (pcDNA-V5-DEST). Protein was harvested two days post transfection for the detection of activated Ras via a pull down assay for the Ras binding domain (RBD) of Raf, and detected by WB.

#### Glucose uptake

3T3-L1 cells that constitutively express Ad36 E4orf1 or a mutated E4orf1 (deleted PBM; ΔPBM) were generated as previously described [9], plated on 12-well plates, and basal 2DG uptake was determined as described in T&A.

### Experiment 5: Does E4orf1 modulate glucose disposal in preadipocytes, adipocytes, myoblasts or hepatocytes?

In separate experiments, 3T3-L1 preadipocytes or adipocytes, C2C12 myoblasts or HepG2 hepatocytes were transfected with V-5 tagged plasmids expressing E4orf1 (pcDNA-V5-AD36-E4orf1) or a null vector (pcDNA-V5-DEST) as described below.

#### 3T3-L1 preadipocytes

3T3-L1 preadipocytes were plated in 12-well plates and transfected the next day. Two days post transfection, basal and insulin (100 nM) stimulated 2DG uptake was determined as described in T&A.

#### 3T3-L1 adipocytes

3T3-L1 preadipocytes were differentiated as described in T&A for 6 days. The differentiated adipocytes were transfected by electroporation. Two-days post transfection, basal or insulin (100 nM) stimulated 2DG uptake was determined as described in T&A.

#### C2C12 myoblasts

C2C12 cells were transfected by electroporation, and two-days post transfection, basal or insulin (100 nm) stimulated 2DG uptake was determined as described in T&A.

#### HepG2 hepatocytes

HepG2 cells were transfected using the AMAXA nucleofector device (Lonza, #610-10-1) and plated in 96-well plates. Forty hours post-transfection, cells were switched to serum free medium for 5 hours. Next, they were exposed to serum free-glucose free media (Invitrogen, #11966) with 0 or 10 nM insulin for 4 hours. Glucose concentration in the media was determined using a glucose oxidase assay (Raichem, #R80038). Glucose values were adjusted to protein concentration as measured by bicinchoninic-acid assay (BCA) (Sigma Aldrich # B9643, #C2284) after solubilization of cells with 0.05% sodium dodecyl sulfate (SDS).

### Techniques and Assays

#### A. Cell Culture

Maintenance media: 3T3-L1 cells were obtained from American Type Culture Collection (ATCC #CCL-92-1, Mannassas, VA) and maintained in high glucose Dulbecco's Modified Eagle Medium (DMEM) (Invitrogen, #11995), 10% normal calf serum (#SH30072.03, Hyclone) and an antibiotic-antimyotic agent (1%) (Sigma Aldrich #A5955). 3T3-L1 E4orf1 clone was maintained in Tet-free fetal bovine serum (Clonetech, #631101) with 0.25 µg/mL puromycin and 0.05 µg/mL hygromycin (Invitrogen, #10687-010).

Adipogenic differentiation: Two days post confluence, 3T3-L1 preadipocytes plated in 10 cm dishes were induced to differentiate by adding DMEM containing 10% FBS (#SH30071.03, Lot: ASL30786, Hyclone), 20 ng/mL insulin, 115 ng/mL isobutylmethylxanthine, and 0.39 ng/mL dexamethasone. Forty-eight hours after the induction, cells were switched to maintenance media of DMEM containing 10% FBS, and 5 ng/mL insulin.

C2C12 myoblasts were obtained from Dr. Kenneth Eilertsen at the Pennington Biomedical Research Center, LA. Cells were maintained in DMEM, 10% fetal calf serum, and an antibiotic-antimycotic agent (1%).

HepG2 cells were obtained from Dr. Jianping Ye at the Pennington Biomedical Research Center, LA. They were maintained in DMEM, 10% fetal calf serum, and an antibiotic-antimycotic agent (1%).

#### B. Virus preparation

Ad36 was obtained from American Type Culture Collection (ATCC # VR-913) and propagated in A549 cells after plaque purifying three times as described [13], [14]. Viral titers were determined by plaque assay and cell inoculations were expressed as plaque forming units (PFU) per cell [13], [14].

#### C. siRNA Transfection

3T3-L1 preadipocytes were plated into 12-well plates (80,000 cells/well) and transfected the next day with Lipofectamine 2000. A ratio of 2 µL lipofecatmine and 100 pmol Ad36 E4orf1 siRNA or NT siRNA was used for transfection, which produced 60% knockdown of E4orf1 expression. Knockdown was confirmed two days post transfection by harvesting RNA and detecting E4orf1 mRNA via quantitative real time PCR. The following E4orf1 siRNA sequence was custom designed by Ambion:

Sense siRNA Strand (5′→3′): GAGAGUGAUUUUUCCUUCATT

Antisense siRNA Strand (5′→3′): UGAAGGAAAAAUCACUCUCTC

#### D. Creation of stable E4orf1 inducible cell line

TetOn Advanced system (Clonetech, #630930) was used to create a stable inducible E4orf1 expressing cell line. Early passage 3T3-L1 cells were co-transfected with the TetON plasmid and a linear puromycin marker (Clonetech #ST0207), and stable transfectants were selected using 1 µg/mL puromycin. Selected clones were screened for TetON integration using transient transfection of p-Luc-TIGHT. A luciferase assay (Promega, #E1501) was conducted following doxycycline treatment to identify the clone with the highest luciferase expression. That clone was then co-transfected with a linear hygromycin marker (Clonetech, #ST0206) and the gene of interest plasmid, either pTRE-TIGHT (null vector control) or pTRE-E4orf1-TIGHT, and double stable transfectants were selected with a maintenance dose of 0.25 µg/mL puromycin and a selection dose of 0.1 µg/mL hygromycin. After 15 days selection, several clones were screened for E4orf1 expression at different doses and time points post doxycycline treatment. RNA was harvested 16, 24, and 48 hours post treatment with 0, 500, 750, and 1,000 ng/ml doxycycline. E4orf1 expression levels were detected via quantitative real time PCR. The optimal dose and time point with maximal E4orf1 expression was determined as 1,000 ng/mL doxycycline, at 24 hours post treatment.

#### E. Western Blotting

For all WB involving Ad36 E4orf-1-V5-pcDNA or pcDNA-V5-DEST transfection, cells were harvested 2 days post transfection in RIPA buffer with protease and phosphotase inhibitors. Protein concentration was determined with a BCA assay. SDS-PAGE was performed and proteins were transferred to PVDF membrane. Twenty µg protein was loaded in a 7% gel for detection of Glut4 with monoclonal antibody (R&D Systems # MAB1262) at 1∶1000 dilution. Fifteen µg protein was loaded in to a 10% gel for determination of Ras (Cell Signaling #3965S) at 1∶1000 dilution. To confirm successful transfection of V5-tagged E4orf-1, V5 antibodies were used at 1∶1000 dilution. Ten percent gels were loaded with 15 µg protein and probed for p-AKT and total AKT (1∶1000) (Cell Signaling #'s 9271 and 9272, respectively). For detection of Glut 1, 7.5% gels were loaded with 30 µg protein and probed with 1∶200 dilution of Glut1 antibody (Santa Cruz Biotechnologies, # sc-1605). Adiponectin was detected using 1∶1,000 dilution of antibody (Millipore # MAB3608) after 15 µg protein was loaded in 15% gel. A 1∶1000 dilution of beta tubulin antibody (Santa Cruz Biotechnologies, # sc-9104) was used after Ras activation assay for housekeeping. For all other proteins, β-actin was used as a loading control (Santa Cruz # sc-69879)(1∶500 dilution). Densitometry was conducted using Image J software by the National Institutes of Health, and all graphs are represented as the ratio of the protein of interest to β-actin.

#### F. Ras activation assay

3T3-L1 preadipocytes were transfected with Ad36 E4orf-1-V5-pcDNA, Ad36 E4orf-1ΔPBM-V5-pcDNA or pcDNA-V5-DEST using a 3∶1 ratio of Lipofectamine to plasmid DNA, and harvested two days post transfection for the Ras activation assay. Ras Activation Kit (#17-218, Upstate) was used according to manufacturer's protocol. After pull-down, 3% of the total whole cell lysate and the slurry was loaded to a 15% gel, and subjected to SDS-PAGE. After transfer to PVDF membrane, the Anti-RAS CLONE10 antibody provided with the Upstate kit was used to detect activated Ras at the suggested dilution. Using NIH Image J, total Ras was normalized to β-actin expression, and the ratio of activated to normalized total Ras is presented in the figures.

For detection of the specific isoforms of Ras activated by E4orf1, the 3T3-E4 inducible clone and pTRE empty vector clone were induced with 1,000 ng/mL doxycycline for 24 hours. After the Ras activation assay (#17-218, Upstate), four separate gels were loaded with 6% of the whole cell lysate for determination of total Ras and the slurry for determination of activated Ras. Each WB was probed with either 1∶200 dilution of H-, N-, or K-Ras antibodies (Santa Cruz # sc-520, sc-519, and sc-30, respectively) for the detection of each isoform, or the Anti-RAS CLONE10 antibody for determination of total Ras activation. Using NIH Image J, total Ras was normalized to β-actin expression, and the ratio of activated to normalized total Ras is presented in the figures.

#### G. Dlg1/E4orf1 interaction

HEK 293 cells were co-transfected with plasmid expressing Dlg1 (GW1-CMV-HA-Dlg1 provided by Dr. Ronald Javier, Baylor College of Medicine, Houston, TX) and either one expressing E4orf1 (pcDNA-V5-Ad36 E4orf1) or a control plasmid (pcDNA-V5-DEST) (Invitrogen #12489-019). Cells were harvested 48 hours later and lysed in buffer containing Tris-HCl 50 mM pH 7.5, 1% Triton X-100 150 mM NaCl and complete anti-protease (Sigma Aldrich, #P8340) anti phosphatase inhibitor cocktail (Thermo Scientific #78420). One mg of total protein was used to immunoprecipitate Dlg1 with 5 µg of anti-HA antibodies (Santa Cruz, #sc-7392). Immonoprecipitated protein complexes were extensively washed and loaded on SDS-PAGE gel. Membranes were probed with anti-HA and anti-V5 antibodies (Invitrogen, #46-0705; 1∶1000).

#### H. Plasmid Preparation and Transfection

The plasmid pcDNA-V5-E4orf1 was cloned using Invitrogen GateWay system that included two steps. 1) Direct TOPO cloning of Ad36 E4orf1 cDNA and Ad36 E4orf1ΔPBM into pENTR-D TOPO vector (Invitrogen #K2400-20) according to manufacturer's manuals. 2) pENTR-E4orf1 vectors from first step was used for recombination with pcDNA-V5 vector (Invitrogen #12489-019) using LR Clonase enzyme (Invitrogen # 11791-019) to obtain pcDNA-V5-E4orf1 which was used for cell transfection.

3T3-L1 preadipocytes were transfected with Lipofectamine (Invitrogen #18324-012). Cells were plated into 12 well plates (80,000 cells/well), and transfected the next day with a 3∶1 ratio of lipofectamine to plasmid DNA according to the manufacturer's instructions. Transfection was verified via protein harvest and WB for the detection of V5- tagged E4orf1 with V5 antibodies.

3T3-L1 adipocytes were transfected on day 6 post adipogenic induction. After trypsinizing with 0.25% trypsin, cells were counted and transfected by electroporation with AMAXA kit nucleofection solution L (Lonza, #VCA-1005) according to the manufacturer's protocol except that each cuvette contained 2.5 million cells and was plated to two wells of a 12 well plate. The next day after the cells had settled on the plate, media was gently removed and replaced with fresh media to remove any remaining transfection solution and the cells that did not survive transfection. Transfection was verified via protein harvest and WB for the detection of V5- tagged E4orf1 with V5 antibodies.

HepG2 cells were transfected using the AMAXA nucleofector device, Cell Line Nucleofector Kit V (Lonza #VACA-1003) with either pcDNA-V5-Ad36-E4orf1 or pcDNA-V5-DEST plasmids and plated in 96-well plates. Nearly confluent HepG2 cells were trypsinized, counted, and 2 µg of plasmid and 2 million cells were used per cuvette. Nucleofector program T-028 was used for transfection, and after resuspension 40,000 cells per well were plated on 96-well plates for the glucose output assay. Transfection was verified via protein harvest and WB for the detection of V5- tagged E4orf1 with V5 antibodies. Glucose output in media was determined using a glucose oxidase assay (Raichem #R80038) and normalized to protein content for each well, which was determined by BCA.

#### I. Quantitative real-time PCR

E4orf1 was detected using quantitative real time PCR (qRT-PCR). RNA was harvested and isolated using a RNA Mini Easy Kit (Qiagen, #74104), and cDNA was synthesized via RT PCR (Applied Biosystems # 4368814) according to manufacturer's instructions. An Ad36 E4orf1 FAM nonflourescent primer-probe was custom designed and synthesized (Integrated DNA Technologies). The E4orf1 primer probe combination is as follows:

Probe 5′-/56-FAM/TGC TGC TCT/ZEN/TTA ACC ACA CGG ACC G/3IABkFQ/-3′

Primer 1 5′-CCC TCG CGG ACATAC AAA A-3′

Primer 2 5′-GCC GGG AGA AGA CAT GAT CTC-3′

One hundred ng of cDNA was loaded per well in duplicate for detection of E4orf1. GAPDH (glyceraldehyde phosphate dehydrogenase) primer probe was used as a housekeeping gene (Applied Biosystems, assay ID# Mm99999915_g1), and 2 ng per well was loaded in duplicate for each sample. Taqman Universal PCR Mix was used for both genes according to manufacturer's instructions (Applied Biosystems #4304437). Expression was detected with the Applied Biosystems 7900 Sequence Detection System and the ΔΔCt method.

#### J. Glucose uptake

Cells in 12 well plates were serum starved for 2 hours, and then washed 2× with PBS before adding 450 µL KRP (136 mM NaCl, 4.7 mM KCl, 10 mM NaPO4, 0.9 mM CaCl2, 0.9 mM MgSO4) with or without 100 nM insulin (Sigma Aldrich, #15500), depending on the experiment, for 15 minutes. Two to three wells were treated with KRP plus 100 nM cytochalasin (Sigma Aldrich, #C6762) for subtraction of nonspecific glucose uptake. Fifty µL of 10× isotope solution was then added to each well for a final concentration of 100 nM cold 2-deoxy glucose and 0.5 µCi/mL [3H]-2-Deoxyglucose (PerkinElmer #NEC720A250UC) for 5 minutes. Immediately after the 5 minute incubation, cells were washed in ice-cold PBS. 500 µL of 0.05% SDS was then added to each well, and after incubation of cells for 30 min at 37°C, 450 µL of cell lysate was added to a scintillation vial and the remaining 50 µL was used for protein determination via BCA assay. Samples were read on a Beckman scintillation counter the following day and readings were normalized to protein content of each well.

### Statistical Analyses

The experiment to knock down E4orf1 with siRNA was repeated three times. Experiments to determine 2DG uptake in 3T3-L1 adipocytes and glucose output in HepG2 cells following E4orf1 transfection were repeated twice. All assays were performed with a minimum of three biological replicates. For all metabolic assays, 8–12 biological replicates were used, and normalized to protein content. For WB assays, three biological replicates were used, and using densitometry, the expression of protein of interest was normalized to a housekeeping protein. Normalized protein values were compared between groups. AKT phosphorylation is presented as a ratio of p-AKT to total AKT. Activated Ras is presented as a ratio of activated Ras to total Ras normalized to β-actin. Group means were compared to test the hypotheses, by using a one-way Student's t-test. Significance was considered at p<0.05.

## Results

### Experiment 1: Ad36 ‘requires’ E4orf1 for glucose uptake

**Figure 1:**
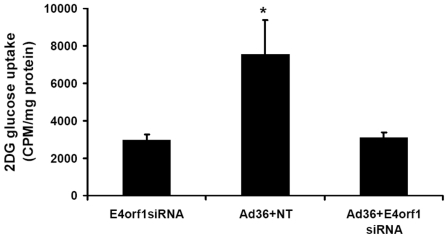
Ad36 ‘requires’ E4orf1 for glucose uptake in 3T3-L1 cells. 3T3-L1 were transfected using lipofectamine with 100 pmol non-targeting (NT) siRNA or E4orf1 siRNA. E4siRNA transfected cells were immediately Mock infected or Ad36 infected (5 PFU/cell), and NT transfected cells were infected with Ad36 as positive control. 2DG uptake was determined and protein was harvested 2 days post transfection/infection. Ad36 infected 3T3-L1 with NT siRNA transfection had significantly greater glucose uptake than Mock-infected cells with E4orf1 siRNA (p = 0.007). When E4orf1 expression was knocked down with E4orf1 siRNA, the effect of Ad36 on glucose uptake was inhibited.

To establish E4orf1 as a candidate protein for glucose uptake, we first investigated if Ad36 requires E4orf1 for glucose uptake, by knocking down E4orf1 expression. E4orf1 siRNA, but not NT siRNA, knocked down the expression of E4orf1 mRNA by 60% in Ad36 infected cells, as determined by real time qRT-PCR (data not shown). Predictably, cells infected with Ad36 and transfected with NT RNA, increased 2DG uptake by nearly 2.5 fold compared to mock infected cells transfected with E4orf1 siRNA (p = 0.007; Figure 1). A knockdown of E4orf1 with siRNA (Ad36+E4orf1 siRNA group) almost entirely abrogated the effect of Ad36 on 2DG uptake (Figure 1), indicating that E4orf1 is essential for Ad36 to increase glucose uptake.

### Experiment 2: E4orf1 is ‘sufficient’ to up-regulate cellular glucose uptake. Also, E4orf1 up-regulate the Ras pathway

**Figure 2:**
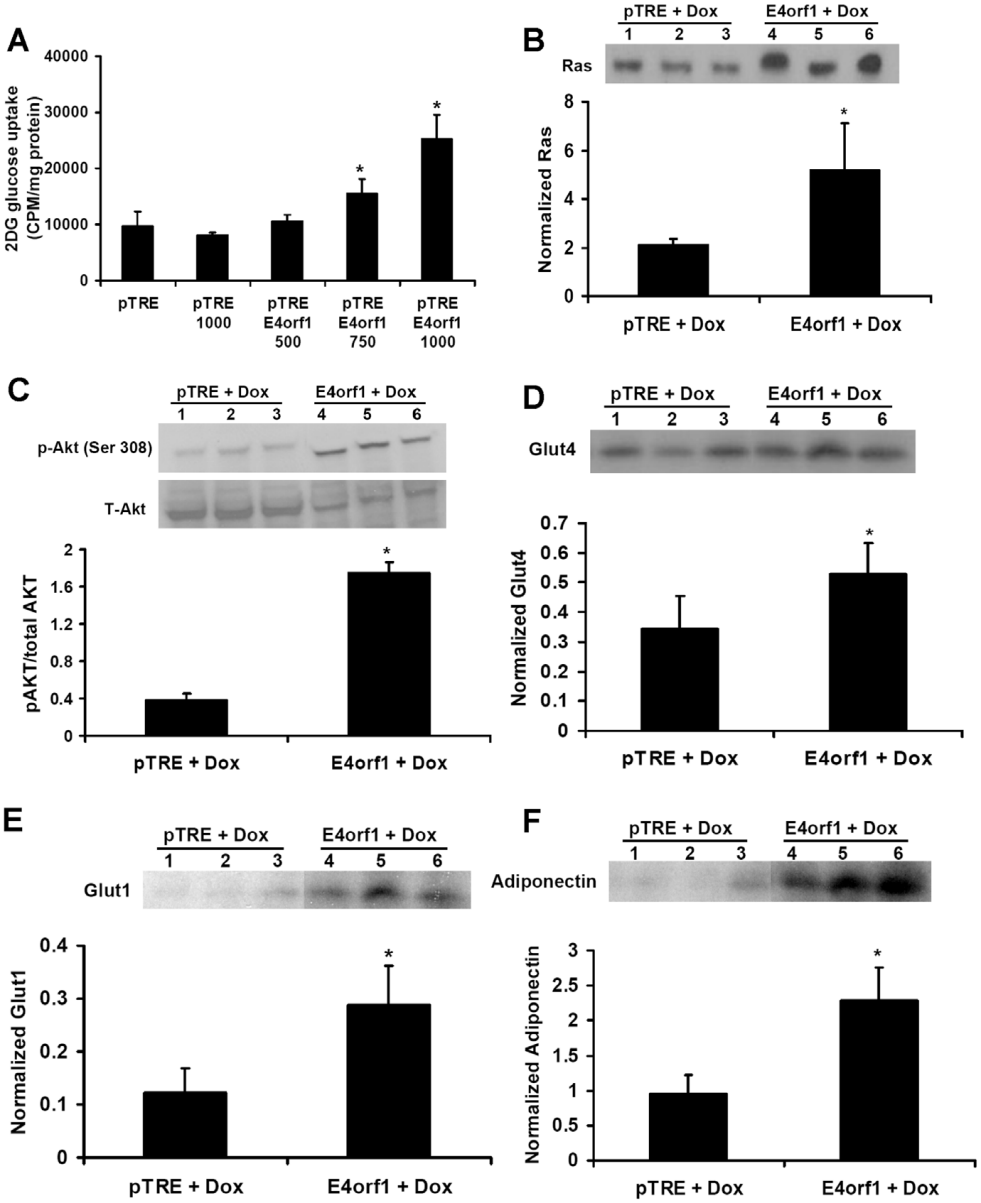
E4orf1 is ‘sufficient’ to up-regulate cellular glucose uptake and to up-regulate the Ras/PI3k pathway. 3T3-E4(pTRE E4orf1) and pTRE Null (pTRE) cell lines were treated with varying doses of Doxycycline for 24 h, and a dose-dependent 2DG uptake was determined. Protein was also harvested from 3T3-E4 and pTRE Null treated with 1,000 ng/ml Doxycycline for 24 hours to determine signaling changes with E4orf1 expression. **A**) E4orf1 is sufficient to induce a dose-response increase in glucose uptake. 3T3-E4 and pTRE Null control cell lines were treated with 500, 750, or 1,000 ng/ml Doxycycline to induce different levels of E4orf1 expression, 2DG uptake was determined. Compared to pTRE, the E4orf1 expressing cell had significantly greater glucose uptake when treated with 750 and 1,000 ng/ml Doxycycline (p * = 0.007 and ** = 2.6×10^−7^, respectively). **B**) Ras WB and densitometry normalized to β-actin for 3T3-E4 and pTRE Null treated with 1,000 ng/ml Doxycycline for 24 hours. Ras expression was significantly higher in the 3T3-E4 cell line (p = 0.05). **C**) p-AKT and AKT WB and densitometry expressed as ratio of p-AKT to total AKT in 3T3-E4 and pTRE Null treated with 1,000 ng/ml Doxycycline for 24 hours. p-AKT is significantly higher in the 3T3-E4 cell line (p = 3.54*10^−5^). **D**) Glut4 WB and densitometry normalized to β-actin for 3T3-E4 and pTRE Null treated with 1,000 ng/ml Doxycycline for 24 hours. Glut4 expression was significantly higher in the 3T3-E4 cell line (p = 0.05). **E**) Glut1 WB and densitometry normalized to β-actin for 3T3-E4 and pTRE Null treated with 1,000 ng/ml Doxycycline for 24 hours. Glut1 expression was significantly higher in the 3T3-E4 cell line (p = 0.01). **F**) Adiponectin WB and densitometry normalized to β-actin for 3T3-E4 and pTRE Null treated with 1,000 ng/ml Doxycycline for 24 hours. Adiponectin expression is significantly higher in the 3T3-E4 cell line (p = 0.006).

3T3-L1 cells that inducibly and stably express Ad36 E4orf1 (3T3-E4 cells) when treated with different doses of doxycylcine (500, 750, or 1,000 ng/ml), induced different expression levels of E4orf1 as determined by qRT-PCR (data not shown). 2DG uptake was nearly identical in pTRE cells with or without exposure to 1000 ng/mL doxycycline (Figure 2A). However, compared to pTRE cells, exposure to doxycline dose-dependently induced 2DG uptake in 3T3-E4 cells (Figure 2A). Compared to pTRE cells, 3T3-E4 cells treated with 750 and 1,000 ng/mL increased glucose uptake by 1.5 and 2.5 fold, respectively. Proteins harvested from pTRE cells and 3T3-E4 cells exposed to 1,000 ng/mL doxycyclin were used for determining cell signaling.

Previous studies indicated that Ad36 up-regulates Ras/PI3K/Glut4 pathway, and Glut1 and adiponectin – an insulin sensitizing adipokine, to increase cellular glucose uptake [1]–[3]. In concordance, E4orf1 expression also up-regulates the Ras/PI3K/Glut4 pathway, as indicated by significantly greater abundance of Ras, p-AKT, Glut4, Glut1 and adiponectin (Figure 2 B–F). As described below, we next determined if E4orf1 ‘activates’ Ras.

### Experiment 3: E4orf1 interacts with Dlg1 and activates Ras

Considering the significance of Ras in Ad36-induced glucose uptake [2], [3], we further investigated the interaction of E4orf1 with Ras. Based on their systematic investigation of Ad 9 E4orf1 interaction with host proteins, Frese et al [10], [11] proposed that Ad9 E4orf1 binds to Dlg1 through its PBM. This binding triggers the resulting complex to translocate to the plasma membrane to promote Ras-mediated PI3K activation, for which the PBM region of Ad9 E4orf1 is necessary. Modeled on this information, Experiment 3 determined if Ad36 E4orf1 complexes with Dlg1 and whether E4orf1 activates Ras. The next experiment (Experiment 4) determined if PBM of Ad36 E4orf1 is required for Ras activation and glucose uptake.

**Figure 3:**
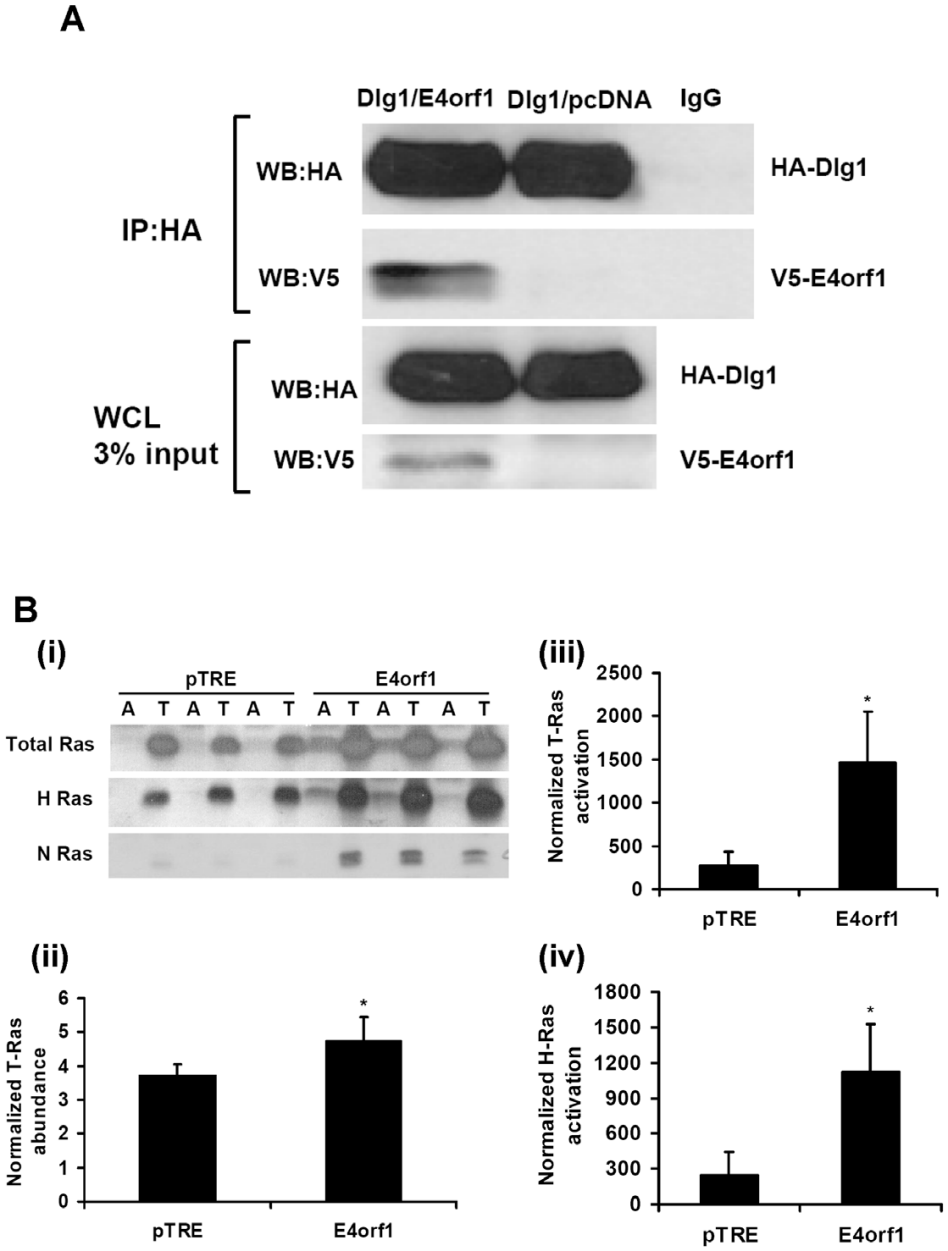
E4orf1 interacts with Dlg1 and activates Ras. HEK293 were co-transfected with GW1-CMV-HA-Dlg1 and either pcDNA-V5-Ad36 E4orf1 or a control plasmid (pcDNA-V5-DEST), and a pull down for HA was conducted and loaded to SDS-PAGE. WB were probed with V5 antibody to detect Dlg1 and E4orf1 interaction. Also, 3T3-E4 and pTRE Null cell lines were treated with 1,000 ng/ml Doxycycline, and a Ras activation assay was conducted. Following pull down, activated slurry and 6% of the whole cell lysate were loaded to SDS-PAGE and probed with total Ras antibodies, or H-, N-, or K-Ras specific antibodies. Blots were also probed with β-actin, and densitometry was expressed as ratio of activated Ras to normalized total Ras. **A**) WB show HA-Dlg1 and V5E4orf1 in the same complex after IP, indicating the binding of Ad36 E4orf1 with Dlg1. WB also shows the presence of Dlg1 in the whole cell lysate (WCL). As expected, V5-E4orf1 is absent in pcDNA-V5-DEST group after IP for HA-Dlg1. **B**. (i) WB for total, H-, and N-Ras activation. T = total (6% WCL) and A = Activated slurry. (ii) Total Ras abundance normalized to β-actin was significantly more in 3T3-E4 compared to pTRE Null vector cell line (p = 0.04). (iii)Total Ras activation densitometry, expressed as ratio of activated to normalized total Ras. Total Ras activation was significantly greater in 3T3-E4 compared to pTRE Null (p = 0.01). (iv) H-ras activation densitometry, expressed as ratio of activated H-Ras to normalized total H-Ras. H-Ras activation was significantly more in 3T3-E4 compared to pTRE Null (p = 0.01).

Co-transfection of HEK293 cells with Dlg1 expressing plasmid (GW1-CMV-HA-Dlg1) and with either plasmid expressing Ad36 E4orf1 (pcDNA-V5-Ad36 E4orf1) or null vector (pcDNA-V5-DEST) showed that Dlg1 and E4orf1 co-immunoprecipitate (Figure 3A). As expected, no E4orf1 precipitated in the IP protein from the group cotransfected with Dlg1 and the null vector. The whole cell lysate shows the HA-Dlg1 and V5-tagged E4orf1 bands, confirming that transfection was successful. This result demonstrated that Ad36 E4orf1 complexes with Dlg1 – similar to the action of E4orf1 of Ad9, which binds to Dlg1 to mediate Ras activation [10], [11].

Next, we determined Ras activation. Ras has three main isoforms, H-, K-, and N-Ras. After exposing 3T3-E4 cells with 1,000 ng/mL doxycycline for 24 hours, total Ras abundance was greater (p = 0.04), Figure 3 B(i & ii)) and total-Ras activation (normalized to total Ras abundance) was about 6-fold higher in the 3T3-E4 cell line compared to the pTRE cell line treated similarly (p = 0.02; Figure 3B (i–iii)). H- and N-Ras isoforms, but not K-Ras isoform were detectable by WB in these cells Figure 3B (i). N-Ras or H-Ras abundance normalized to total Ras were 8 or 1.3 fold greater, respectively (p = 0.0001 and 0.007), for the E4orf1 expressing cells (data not shown). Interestingly, in these preadipocytes, E4orf1 significantly activated only the H-Ras isoform (p = 0.01; Figure 3B (iv)) – the isoform that enhances glycemic control in mice when transgenically overexpressed in adipose tissue [15].

We next identified the region of E4orf1 protein that is required to activate Ras and to up-regulate glucose uptake.

### Experiment 4: E4orf1 requires its PBM for glucose uptake and Ras activation

**Figure 4:**
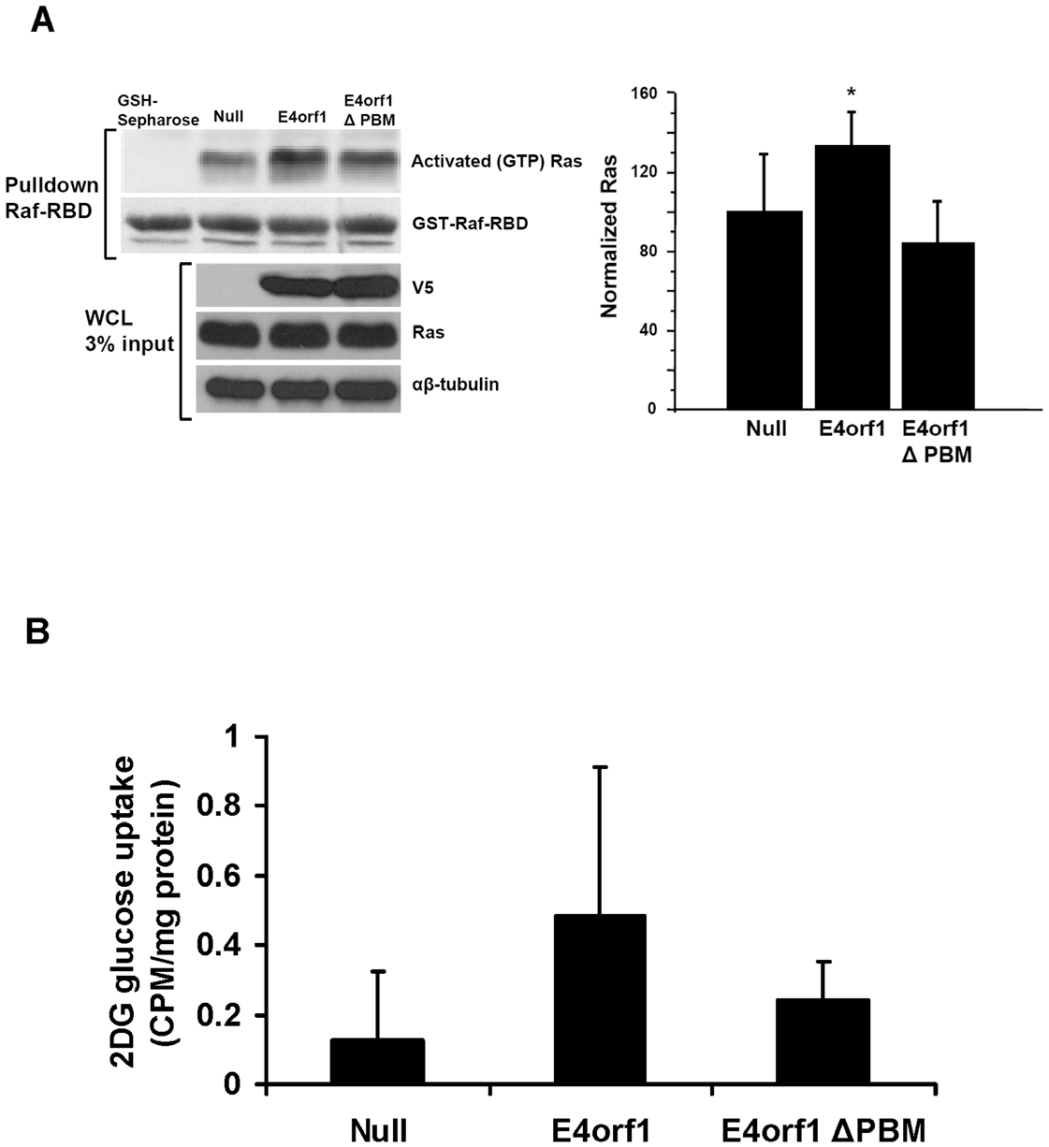
E4orf1 requires its PBM for glucose uptake and Ras activation. 3T3-L1 cells were transfected with V-5 tagged plasmids expressing E4orf1 (pcDNA-V5-AD36-E4orf1), mutant E4orf1 (pcDNA-V5- AD36-E4ORF1ΔPBM) or a null vector (pcDNA-V5-DEST) and a Ras activation assay was conducted the next day. The activated slurry and 3% of whole cell lystate was loaded to SDS-PAGE and probed for Ras. Also 3T3-L1 cells that constitutively express Ad36 E4orf1, a mutated E4orf1 (deleted PBM; ΔPBM) or Null vector were plated in 12 well plates and a 2DG uptake assay was conducted the following day. **A**) WB for Ras activation assay: pcDNA-V5-AD36-E4orf1 activates Ras (p = 0.04), whereas the Null vector (pcDNA-V5-DEST) does not. pcDNA-V5- AD36-E4ORF1ΔPBM did not activate Ras, suggesting E4orf1 depends on its PBM region for Ras activation. Densitometry expressed as ratio of activated Ras to normalized total Ras from 3% WCL WB. **B**) 2DG uptake in constitutive expressing Ad36 E4orf1, a mutated E4orf1 (deleted PBM; ΔPBM) or Null vector normalized to protein: E4orf1 increased glucose uptake compared to Null vector (p = 0.008), and there was no difference in glucose uptake when the PBM region of E4orf1 is mutated.

Ad9 E4orf1 requires its PBM to activate Ras and PI3K [10], [11], and Ad36 E4orf1 requires its PBM to up-regulate PI3K pathway [9]. Hence, this experiment determined if the PBM region of Ad36 E4orf1 is critical for Ras activation and glucose uptake. E4orf1 transfection or constitutive expression significantly increased Ras activation, or increased glucose uptake by nearly 5-fold, respectively (Figure 4 A&B). Mutation in PBM of E4orf1 significantly attenuated the Ras activation or glucose uptake induced by the intact E4orf1 protein (Figure 4 A&B). These data identify the PBM region of Ad36 E4orf1 as an important site for increasing cellular glucose uptake.

The results from experiment 1–4 suggest that Ad36 E4orf1 complexes with Dlg1 to mediate the activation of H-Ras to up-regulate the downstream PI3K/Glut4 pathway and cellular glucose uptake, and the PBM region of E4orf1 is necessary for this action. Next, Experiment 5 identified the cell types responsive to the effect of E4orf1 on glucose disposal.

### Experiment 5: E4orf1 modulate glucose disposal in preadipocytes, adipocytes, myoblasts or hepatocytes

Considering that the glucose uptake by adipose tissue and skeletal muscle and glucose output by the liver contribute to systemic glycemic control, we determined the effect of Ad36 E4orf1on basal and insulin stimulated glucose disposal by cell lines representing these tissues. Glucose disposal in 3T3-L1 preadipocytes or adipocytes, C2C12 myoblasts or HepG2 hepatocytes transfected with E4orf1 expressing plasmid (pcDNA-V5-Ad36 E4orf1) was compared with cells transfected with a null vector (pcDNA-V5-DEST).

**Figure 5:**
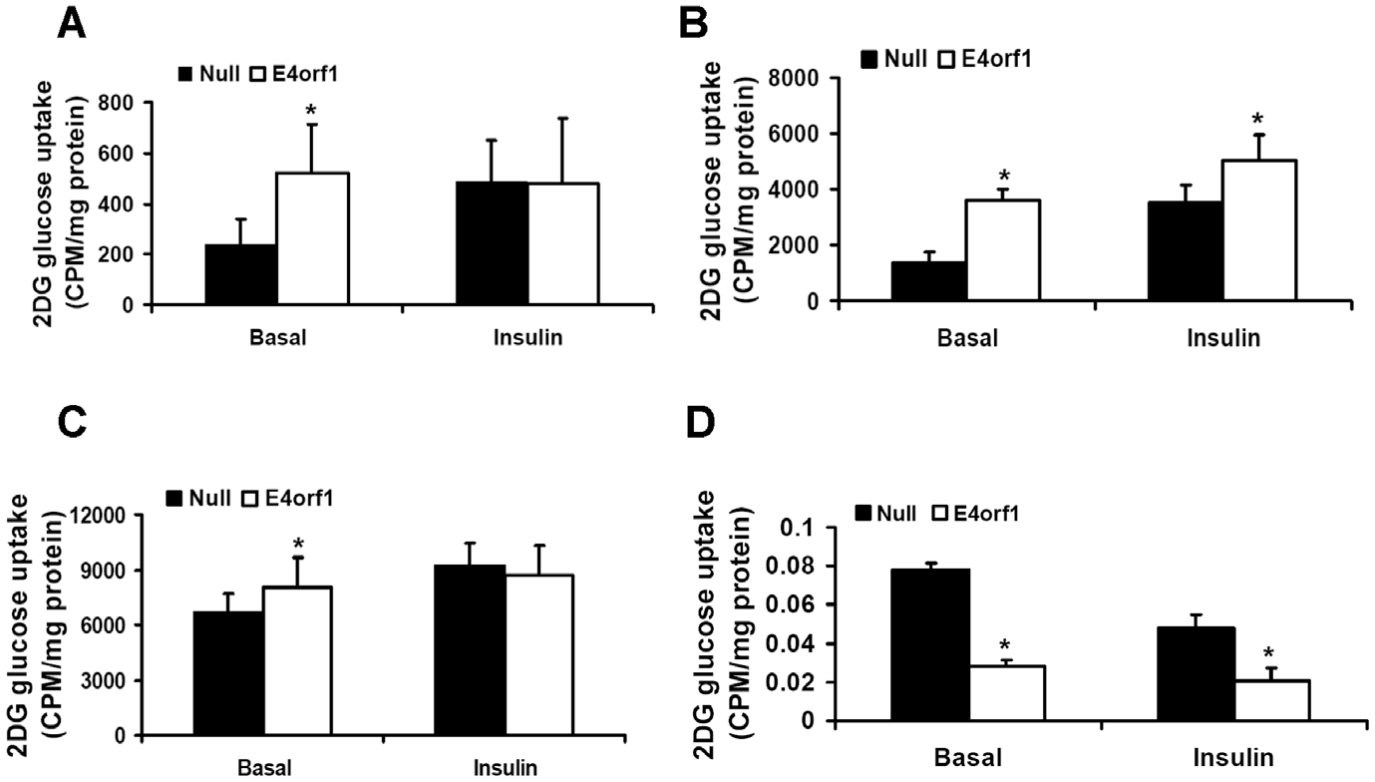
E4orf1 modulate glucose disposal in preadipocytes, adipocytes, myoblasts or hepatocytes. 3T3-L1 preadipocytes, adipocytes, C2C12 myoblasts, and HepG2 cells were transfected with V-5 tagged plasmids expressing E4orf1 (pcDNA-V5-AD36-E4orf1) or a null vector (pcDNA-V5-DEST). 2DG glucose uptake was determined in 3T3-L1 and C2C12 cells, and glucose output was determined in HepG2 cells in basal and insulin (100 nM) stimulated condition. **A**) 2DG uptake in 3T3-L1 preadipocytes: Glucose uptake was normalized to protein a BCA assay arbitrary units (A.U.). Basal pcDNA-V5-AD36-E4orf1 transfected 3T3-L1 glucose uptake is significantly greater than Null pcDNA-V5-DEST vector transfected (p = .0005). **B**) 2DG uptake in 3T3-L1 differentiated adipocytes: Glucose uptake was significantly higher in pcDNA-V5-AD36-E4orf1 transfected adipocytes compared to Null vector (pcDNA-V5-DEST) in the basal and insulin stimulated condition (*p<.0001, **p = .003). **C**) pcDNA-V5-AD36-E4orf1 increases basal glucose uptake in C2C12 myoblasts (p = .05). **D**) pcDNA-V5-AD36-E4orf1 suppresses glucose output in HepG2 cells. pcDNA-V5-AD36-E4orf1 transfected HepG2 cells have significantly lower glucose output in the basal and insulin stimulated (10 nM) condition compared to null vector (pcDNA-V5-DEST) transfected cells (p = 7.3×10^−7^ and .008, respectively).

E4orf1 expression significantly increased basal 2DG uptake in 3T3-L1 preadipocytes, adipocytes and C2C12 myoblasts (Figure 5 A–C). In adipocytes, E4orf1 further increased insulin stimulated 2DG uptake (p = 0.003). In preadipocytes and myoblasts, which are not fully insulin responsive, E4orf1 did not enhance insulin stimulated 2DG uptake.

Although multiple metabolic functions of the liver such as glucose uptake, glycogen synthesis, glycogenolysis, contribute to systemic glycemic control, hepatic glucose output is often uncontrolled due to insulin resistance, and can be a key contributor of high blood glucose in type 2 diabetes [16]. Therefore, we focused on determining the effect of E4orf1 on glucose release by hepatocytes. E4orf1 transfection significantly reduced glucose output by HepG2 cells under basal as well as insulin stimulated conditions (p<0.000001 and <0.001, respectively; Figure 5D).

This experiment suggested that Ad36 E4orf1 may influence glucose disposal by adipose tissue, skeletal muscle and liver.

## Discussion

Ad36 infection improves glycemic control in chow-fed normoglycemic rats and mice [1], [17] and in high fat fed hyperglycemic mice [1]. Natural infection with Ad36 predicts better glycemic control in normoglyemic and diabetic humans [1], [2]. The virus appears to exert its anti-hyperglyemic action by increasing glucose uptake by preadipocytes, adipocytes, and myocytes, and by reducing hepatic glucose output [1]–[3]. Ras/PI3K pathway activation is required for Ad36-induced cellular glucose uptake [2], [3]. These findings are potentially highly significant for developing new treatment approaches for type 2 diabetes and insulin resistance. Particularly, the unique capability of Ad36 to attenuate hyperglycemia despite a continued HF-diet and without a reduction in visceral or subcutaneous adiposity [1] offers a remarkable opportunity to creatively negate the hyperglycemic effects of excess adiposity or dietary fat intake, without the need to reduce it. However, for developing a therapeutic approach, infection with a virus is impractical. Instead, a viral protein that is responsible for the effect could provide a drug ligand or a target. Here we show that E4orf1 is required to mediate the glucose uptake induced by Ad36. Also, E4orf1 is sufficient to promote glucose uptake in preadipocytes, adipocytes, and myoblasts, and to reduce glucose output by hepatocytes.

Ad9 E4orf1, which is 96% homologous to Ad36 E4orf1, mediates Ras activation by complexing with Dlg1 via its PBM [10], which also appears to be the case with Ad36 E4orf1. Ad36 E4orf1 activates Ras and PI3K, the two main signaling components required for Ad36 infection-induced glucose disposal [2], [3]. Ad36 E4orf1 requires its PBM for activating Ras and for up-regulating glucose uptake. Specifically, Ad36 E4orf1 increases the relative abundance and activation of H-Ras isoform.

Conventionally, insulin signaling for glucose disposal could be divided in proximal signaling (the binding of insulin to its receptor followed by the activation of insulin receptor substrates (IRS)1 and IRS 2), and the distal signaling, which includes the activation of PI3K pathway by IRS1 and IRS2, which leads to glucose transporter mediated glucose disposal [1]. E4orf1 appears to activate Ras, to induce the distal insulin signaling pathway. Ras, an important GTP binding protein [18],[19], has been recognized to induce PI3K/AKT pathway[20], or mimic insulin action on glucose transporters Glut4 and Glut1 [21], [22]. In a mouse model, transgenic overexpression of H-Ras in adipose tissue increased insulin sensitivity, and up-regulated adipose tissue Glut4 and Glut1 and glucose uptake even in absence of insulin [15]. Ras-induced glucose disposal was ignored since it plays a negligible role in insulin-stimulated glucose uptake [23]–[25]. Conversely, Ras/PI3K pathway may be very valuable as an alternate pathway to promote cellular glucose disposal, if insulin signaling is impaired. In the absence of functional insulin signaling as in type 2 diabetes or obesity [26]–[32], an agent such as E4orf1 that up-regulates insulin independent glucose disposal through Ras activation may be valuable. Very recently, Ras seems to re-attract attention for its insulin-independent effects on glucose metabolism [33]. Future experiments that knockdown Ras will determine if, like Ad36, its E4orf1 protein also ‘requires’ Ras for promoting glucose disposal. These data provide important information needed to design ligands and therapeutic targets for improving glycemic control.

Although Ras is known as an oncogene, its activation alone is not sufficient to induce tumor formation. For instance, dysregulated focal adhesion kinase is necessary for Ras activation to result in cell transformation [34]. In addition, transgenic over expression of H-Ras in adipose tissue, the specific isoform activated by E4orf1, does not cause tumor formation [15]. Although Ad36 up-regulates the Ras/PI3K pathway, in several experiments lasting up to 7 months, Ad36 infected animals did not develop tumors [13], [14], [17], [35]. Lastly, both Ad36 infection and E4orf1 transfection are unable to induce anchorage independent growth, a marker of cell transformation (unpublished data). Therefore, we hypothesize that E4orf1 is not likely to be oncogenic, although this should be tested more thoroughly *in vivo*.

E4orf1 modulated glucose disposal in pre-adipocytes, adipocytes and, myoblasts indicate that E4orf1 may increase glucose disposal in adipose tissue and skeletal muscle, both important tissues for glucose clearance *in vivo*. Another physiologically relevant effect of E4orf1 is the reduction of glucose output from HepG2 hepatocytes. In the insulin resistant condition, postprandial glucose output from the liver is often uninhibited, contributing to hyperglycemia [16]. Uncontrolled hepatic glucose output is one of the first signs of type 2 diabetes. E4orf1, however, may be able to diminish this hepatic source of blood glucose. Adiponectin - a key insulin sensitizer secreted by adipocytes [36], is a controller of hepatic glucose output [37], [38]. In a previous study, we had postulated that Ad36 up-regulates adiponectin in adipose tissue, which then mediates the reduction in hepatic glucose output in Ad36 infected mice [39]. The current study indicates that E4orf1 increases adiponectin expression in adipocytes, which may secondarily influence hepatic metabolism. In addition, E4orf1 may directly effect glucose output by hepatocytes. A possible direct effect on the liver is also supported by our previous finding that E4orf1 mRNA expression in the livers of Ad36 infected mice positively correlates with their glycemic improvement [39]. These *in vitro* studies indicate that Ad36 E4orf1 may improve glycemic control *in vivo* through adipose tissue, skeletal muscle, and liver- the three main tissues involved in glucose homeostasis.

The *in vivo* glucose uptake induced by Ad36 is not uncontrolled, as evident from a reduction in circulating glucose and insulin levels observed in Ad36 infected mice, without inducing hypoglycemia [39]. *In vivo*, Ad36 appears to reduce insulin required to maintain glycemic control, indicating an ‘insulin sparing effect’ of the virus [39]. E4orf1 may share this insulin sparing effect of Ad36. In presence of insulin, E4orf1 induced glucose uptake in adipocytes was significantly greater, but not additive.

The ability of E4orf1 to particularly influence basal glucose disposal has additional significance. Most of the currently available anti-diabetic agents are either mimetics, sensitizers, or secretagogues of insulin, which employ insulin signaling pathway for their action. However, insulin resistant states such as obesity or diabetes are often associated with impaired insulin signaling [26]–[32], which may limit the efficacy of such drugs. Therefore, the potential of E4orf1 to up-regulate glucose disposal without insulin stimulation may be valuable for developing more effective anti-diabetic drugs.

We previously reported that in animal models, Ad36 is adipogenic [13], [14], [35] and yet, it improves systemic glycemic control [1], [17] . In humans, natural Ad36 infection is associated with obesity [40]–[44], as well as better glycemic control [1] and lower hepatic steatosis [45] – a marker of insulin resistance. *In vitro* studies show that Ad36 E4orf1 is necessary and sufficient to induce adipogenesis [9]. Taken together, these data suggest that Ad36 E4orf1 protein induces adipogenesis as well as improves glycemic control – an effect that is reminiscent of the action of anti-diabetic agents Thiazolidinediones (TZDs) [46]. However, our very recent data show that adipogenic effect of Ad36 could be successfully uncoupled from its effect on glucose disposal (unpublished data). Given the undesirable role of excess adiposity in glycemic control, these findings increase the potential significance of anti-hyperglycemic action of Ad36. While it is likely that the adipogenic effect of E4orf1 could also be uncoupled from its effect on glucose disposal, it remains unknown at this time.

In conclusion, Ad36 E4orf1 protein enhances glucose disposal in cell types from key tissues involved in glucose homeostasis. Additional studies are needed to further elucidate the molecular interactions of E4orf1 and to determine its effect on glycemic control *in vivo*. Particularly, similar to the action of Ad36, if E4orf1 improves glycemic control without reducing dietary fat intake or body fat, and independent of proximal insulin signaling, the protein would be highly valuable to develop novel anti-diabetic agents that mimic its action.
